# Might radiation therapy in addition to chemotherapy improve overall survival of patients with non-oligometastatic Stage IV non-small cell lung cancer?: Secondary analysis of two prospective studies

**DOI:** 10.1186/s12885-016-2952-3

**Published:** 2016-11-21

**Authors:** ShengFa Su, YinXiang Hu, WeiWei Ouyang, Zhu Ma, QingSong Li, HuiQin Li, Yu Wang, XiaoHu Wang, Tao Li, JianCheng Li, Ming Chen, You Lu, YuJu Bai, ZhiXu He, Bing Lu

**Affiliations:** 1 Department of Thoracic Oncology, Affiliated Hospital of Guizhou Medical University, and Guizhou Cancer Hospital, Guiyang, 550004 China; 2 Teaching and Research Section of Oncology, Guizhou Medical University, Guiyang, 550004 China; 3 Department of Radiation Oncology, Gansu Cancer Hospital, Lanzhou, 730050 China; 4 Department of Radiation Oncology, Sichuan Cancer Hospital, Chengdu, 610041 China; 5 Department of Radiation Oncology, Fujian Provincial Cancer Hospital, Fuzhou, 350013 China; 6 Department of Radiation Oncology, Zhejiang Cancer Hospital, Hangzhou, 310022 China; 7 Department of Thoracic Oncology and State Key Laboratory of Biotherapy, Cancer Center, West China Hospital, Sichuan University, Chengdu, 610041 China; 8 Department of Oncology, Affiliated Hospital of Zunyi Medical College, Zunyi, 563003 China; 9 Tissue Engineering and Stem Cell Research Center of Guizhou Medical University, Guiyang, 550004 China

**Keywords:** Non-small cell lung cancer, Non-oligometastase, Thoracic three-dimensional radiotherapy, Overall survival

## Abstract

**Background:**

The role of radiation therapy in addition to chemotherapy has not been well established in non-oligometastatic Stage IV non-small cell lung cancer (NSCLC). We aimed to investigate overall survival (OS) of non-oligometastatic Stage IV NSCLC treated with chemotherapy with concurrent radiation to the primary tumor.

**Methods:**

Eligible patients were screened from two prospective studies. Oligometastatic and non-oligometastatic NSCLC were defined as having < 5 and ≥5 metastatic lesions, respectively. Prognostic factors for OS were identified by using univariate and multivariate analysis. Landmark analysis and propensity-score matching (PSM) were each performed to further adjust for confounding.

**Results:**

A total of 274 patients were identified as the study cohort: 183 had non-oligometastatic disease. For all 274 patients, those who received a radiation dose ≥63 Gy to the primary tumor and had oligometastatic disease had better OS (*P* < 0.001 and *P* = 0.017, respectively). When patients were subdivided into those with oligometastatic or non-oligometastatic disease, a radiation dose ≥ 63 Gy remained a significant prognostic factor for better OS. For non-oligometastatic patients, multivariate analysis showed that receiving ≥63 Gy radiation, having a GTV <146 cm^3^, having response to chemotherapy, and having stable or increased post-treatment KPS independently predicted better OS (*P* = 0.018, *P* = 0.014, *P* = 0.014, and *P* = 0.001). After PSM in non-oligometastatic patients, a higher radiation dose (≥63 Gy) remained to be correlated with better OS. By landmark analysis, aggressive radiation (≥63 Gy) remained to be correlated with better OS in Pre-PSM cohort (*P* = 0.005) and Post-PSM cohort (*P* = 0.004).

**Conclusions:**

Radiation dose, primary tumor volume, response to chemotherapy and KPS after treatment are associated with OS in patients with non-oligometastatic disease; on basis of effective system chemotherapy, aggressive thoracic radiotherapy may prolong OS.

## Background

Approximately 60% of patients who have been newly diagnosed with non-small cell lung cancer (NSCLC) have distant metastases [1]. The metastatic status of NSCLC are highly variable, which ranges from the presence of a single metastatic lesion to a single organ to multiple lesions in several organs. Hellman et al. [2] proposed a notion is that of oligometastases in 1995, oligometastases is the state in which the patient shows distant metastase are limited in number and locations. In addition to oligometastases, there are many other patients who have extensive and widespread metastases, this metastatic state might be called "non-oligometastases".

In the era of two-dimensional radiotherapy (2D-RT), thoracic radiotherapy has long been used as a palliative care in metastatic NSCLC [3–5]. Recent years, there is increasing evidence showed that patients presenting with oligometastatic NSCLC could benefit from aggressive thoracic radiotherapy beyond palliative irradiation [6–12]. However, there was no consistent definition of oligometastases in these studies.

Although, the term of oligometastatic NSCLC has been used without a consistent definition. In recent years, the general opinion is that patients with 1-5 metastases is oligometastases [7–9, 13]. In general consideration, pharmacotherapy was the main treatment modality, and, radiation to primary tumor not affect survival and should be only given to alleviate symptoms (hemoptysis, cough, pain, and others) in non-oligometastatic Stage IV NSCLC. Thus, research on the treatment modalities for non-oligometastatic NSCLC have mainly focused on pharmacotherapy over the years. Nearly 30% of patients may benefit from molecular targeted therapy [14, 15]. Thus, approximately 70% of patients require system chemotherapy. However, the efficacy of platinum-based combination chemotherapy may have reached a plateau over the past 10-15 years [16, 17].

Radiation to the primary tumor for oligometastatic NSCLC patients, who had <5 metastases, has produced favorable survival outcomes [7–10, 13]. In the early years, published data have indicated that the combination of thoracic radiotherapy and chemotherapy improved the treatment outcomes for limited-stage small-cell lung cancer (SCLC) patients [18, 19]. Recently, a phase 3 randomized controlled trial showed thoracic radiotherapy also improves OS for patients with extensive-stage SCLC who have responded to chemotherapy [20]. The remained question is that whether or not thoracic radiation therapy in addition to chemotherapy is beneficial for overall survival in patients with non-oligometastatic NSCLC (who had ≥5 metastases), like extensive SCLC. Therefore, we collected clinical data from two prospective studies to analyze the survival outcomes of non-metastatic NSCLC patients who had undergone concurrent chemotherapy with three-dimensional radiation therapy (3D-RT) to primary tumor and to determine prognostic factors in this population.

## Methods

### Patient selection

We selected patients presenting with metastaic NSCLC who were enrolled in two prospective studies from January 2003 and May 2012 [11, 12]. The selection criteria were as follows: (1) histologically or cytology confirmed NSCLC; (2) newly diagnosed stage IV disease (staged according to the 2002 system of the American Joint Committee on Cancer); (3) did not receive targeted therapy or immunotherapy during lifetime; (4) age 18-80 years; (5) a Karnofsky Performance Status (KPS) score ≥70%; (6) received at least two chemotherapy cycles and a thoracic radiation dose of at least 36 Gy in 1.8 to 2-Gy fractions; (7) using modern radiation technique (3-dimensional conformal radiation therapy [3DCRT] or intensity modulated radiation therapy [IMRT]) and (8) had complete medical records ( include sex, age, KPS score, tumor histology, N stage, T stage, metastatic status at diagnosis, radiation therapy to primary tumor, treatment response, and having survival outcomes [dead or alive]). This study was reviewed by the ethical review boards in China (Ethics Committee of Guizhou Cancer Hospital, GuiYang, China), and the informed consent was obtained from all patients.

### Definition of oligometastatic and non-oligometastatic disease

The definition of oligometastatic and non-oligometastatic disease in NSCLC varies across studies, which ranges from the presence of a single metastatic lesion to a single organ in some studies to multiple lesions in several organs in others [6, 7, 9, 11, 21, 22]. In our current study, we defined oligometastatic and non-oligometastatic NSCLC according to the number of metastatic lesions; namely that < 5 metastatic lesions was defined as oligometastatic NSCLC, and ≥5 metastatic lesions was defined as non- oligometastatic NSCLC.

### Pretreatment evaluations

All patients underwent fiberoptic bronchoscopy and contrast-enhanced computed tomography (CT) of the chest to evaluate the extent of the primary tumor and regional lymph node status. All patients also underwent bone scintigraphy, contrast-enhanced CT of the abdominal region, and magnetic resonance imaging (MRI) of the brain to detect distant metastases. Positive findings on positron emission tomography (PET) /CT or bone scintigraphy required other additional radiologic confirmation (e.g., MRI or CT of bone). Pretreatment evaluations were to be completed within 2 weeks before treatment was begun.

### Treatment methods

#### Radiotherapy

All select patients received thoracic radiation dose of at least 36 Gy in 1.8-2-Gy fractions. Radiation to primary tumor was implemented by modern techniques (3D-CRT or IMRT). Radiation therapy was given concurrently with the chemotherapy, beginning within 1 week after beginning the first course of chemotherapy. Details of the radiation therapy protocol have been reported previously [11, 12].

#### Chemotherapy

Platinum-based doublet chemotherapy (cisplatin in combination with docetaxel, paclitaxel, pemetrexed, or vinorelbine), given every 21-28 days concurrent with thoracic radiation therapy, was the first-line therapy for all patients. No induction chemotherapy was given prior to radiation. After thoracic radiotherapy was completed, patients demonstrating response or stable disease continued chemotherapy for a total of 4-6 cycles. No maintenance therapy was given.

### Evaluation of treatment response

The treatment responses of tumors, including complete response (CR), partial response (PR), stable disease (SD), and progressive disease (PD), were evaluated according to the Response Evaluation Criteria in Solid Tumors system [23]. To evaluate treatment response of radiotherapy: CR or PR was evaluated as having response, whereas SD or PD as no response. However, to evaluate treatment response of chemotherapy: no change in size or shrinkage in any size of target lesions was evaluated as having response to chemotherapy, whereas increasing in any size of target lesions as no response.

### Statistical analyses

The endpoints of this study was to evaluate overall survival (OS). The OS time was measured from the starting date of treatment. Statistical tests were done with Stata, version 11.2 software. The Kaplan-Meier method was used to calculate the OS, and the curves were compared with log–rank tests. Multivariate Cox regression analysis was used to identify the independent predictors of OS. All significant factors in univariate analysis were further tested in the multivariate analysis. Propensity-score matching (PSM) and landmark analysis requiring a minimum of 8 months OS were each performed in sensitivity studies to further adjust for confounding. All statistical tests were two-sided, and *P* values < 0.05 were considered to indicate statistical significance.

## Results

### Overall treatment outcomes

Totally, 274 eligible patients were included in this study, 91 patients had oligometastatic disease and 183 had non-oligometastatic disease. The follow-up period ranged from 2.0 to 64.0 months; at the time of last follow-up, 15 patients were still alive, and the median survival time for those patients was 40.0 months (range, 12.0–64.0 months). The median OS time for all patients was 13.0 months (95% CI 11.9–14.1), and the OS rates were 50.7% at 1 year, 15.8% at 2 years, and 9.1% at 3 years. OS rates for patients who had received ≥63 Gy thoracic radiation therapy were 55.3% at 1 year, 22.7% at 2 years, and 17.0% at 3 years; corresponding rates for those who received <63 Gy were 46.5%, 9.3%, and 2.5%(χ^2^ = 15.638, *P* < 0.001).

**Fig. 1:**
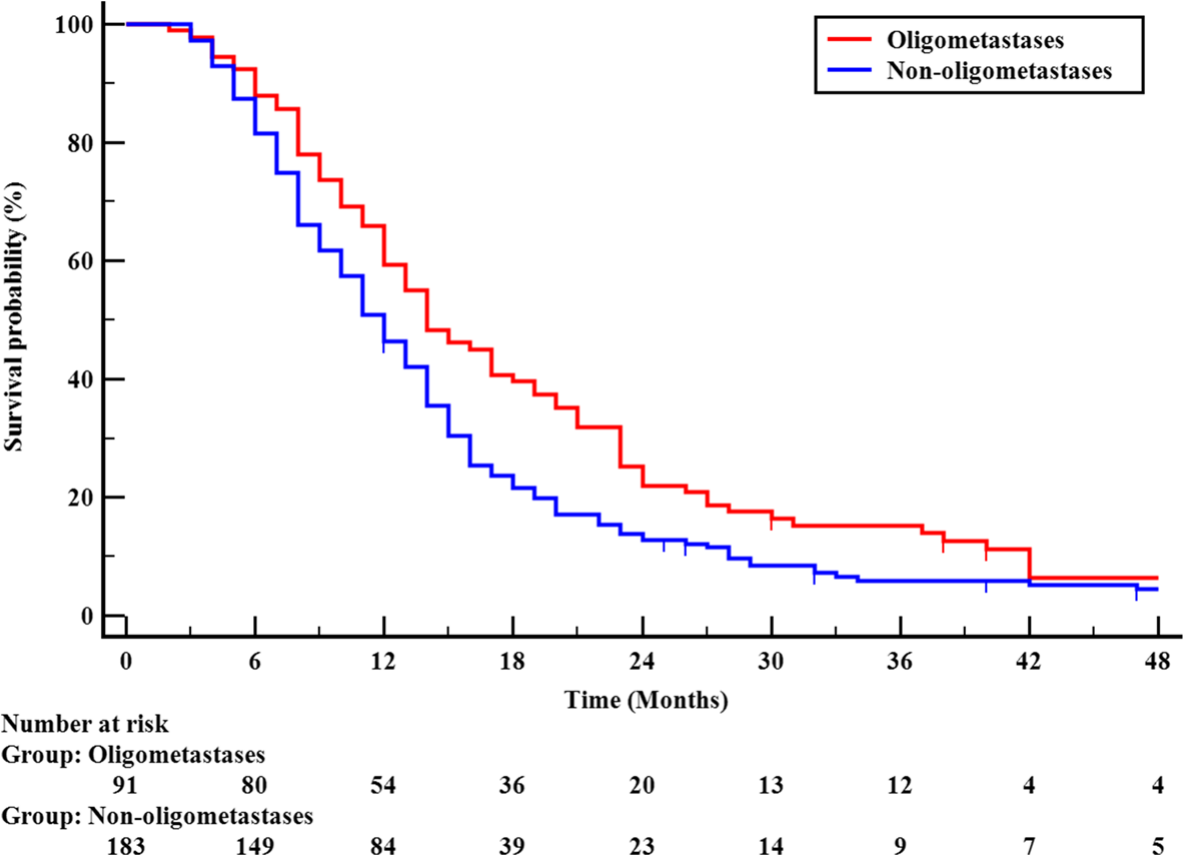
Overall survival grouped by state of metastatic disease (oligometastases and non-oligometastases)

Comparison of OS in patients with oligometastatic disease versus those with non- oligometastatic disease, patients with oligometastatic disease had a better OS. The median survival time (MST) for these two groups were 14.0 months (95% CI, 11.25 – 16.75) and 12.0 months (95% CI, 10.59 – 13.41); the 1-, 2-, and 3-year OS rates were 59.3%, 22.0%, and 15.2% versus 46.4%, 12.7%, and 6.0% (χ^2^ = 5.741, *P* = 0.017), (Fig. 1). When the whole group was subdivided into those with oligometastases (χ^2^ = 6.150, *P* = 0.013) or non-oligometastases (χ^2^ = 8.257, *P* = 0.004), thoracic radiation dose ≥63 Gy remained a prognostic factor for better overall survival.

### Survival analysis of non-oligometastatic Stage IV patients

Seventy-eight patients had metastasis in only one organ: 28 in the bone, 21 in the lung, 23 in the brain, and 6 in other locations. One hundred and five patients had metastasis in two or three organs, the most common site of metastatic disease at diagnosis was the bone (70 of 105 patients), 57 patients had lung metastasis, 51 had metastasis in brain, 16 had metastasis in adrenal, 12 patients had metastasis in distant lymph nodes, six had subcutaneous nodules, and 12 in other locations. Clinical characteristics of non**-** oligometastatic NSCLC patients are listed in Table 1.

**Table 1:** Characteristics of the non-oligometastatic patient cohort before and after PSM

Variable	Pre-PSM Cohort			Post-PSM Cohort		
	<63 Gy	≥63Gy	*P* value	<63 Gy	≥63Gy	*P* value
Total	93	90		59	59	
Gender						
Male	66	59	0.431	47	38	0.065
Female	27	31		12	21	
Age (years)						
<60 years	52	48	0.726	31	27	0.461
≥60 years	41	42		28	32	
KPS Score						
≤80	58	52		32	32	
>80	35	38	0.526	27	27	1.000
Pathological type						
Squamous carcinoma	35	24	0.048	24	20	0.651
adenocarcinoma	47	61		30	35	
Other	11	5		5	4	
T status						
T_1-2_	33	36	0.529	19	22	0.562
T_3-4_	60	54		40	37	
N status						
N_0-1_	11	14	0.463	5	12	0.066
N_2-3_	82	76		54	47	
Response to chemotherapy						
Yes	64	68		43	43	1.000
No	29	22	0.309	16	16	
No. of chemotherapy cycles						
<4	63	32	<0.001	30	30	1.000
≥4	30	58		29	29	
GTV volume (cm^3^)						
<146	44	40	0.697	23	23	1.000
≥146	49	50		36	36	
Metastasis status						
Single organ	37	41	0.430	23	27	0.456
2 to 3 organs	56	49		36	32	

**Fig. 2:**
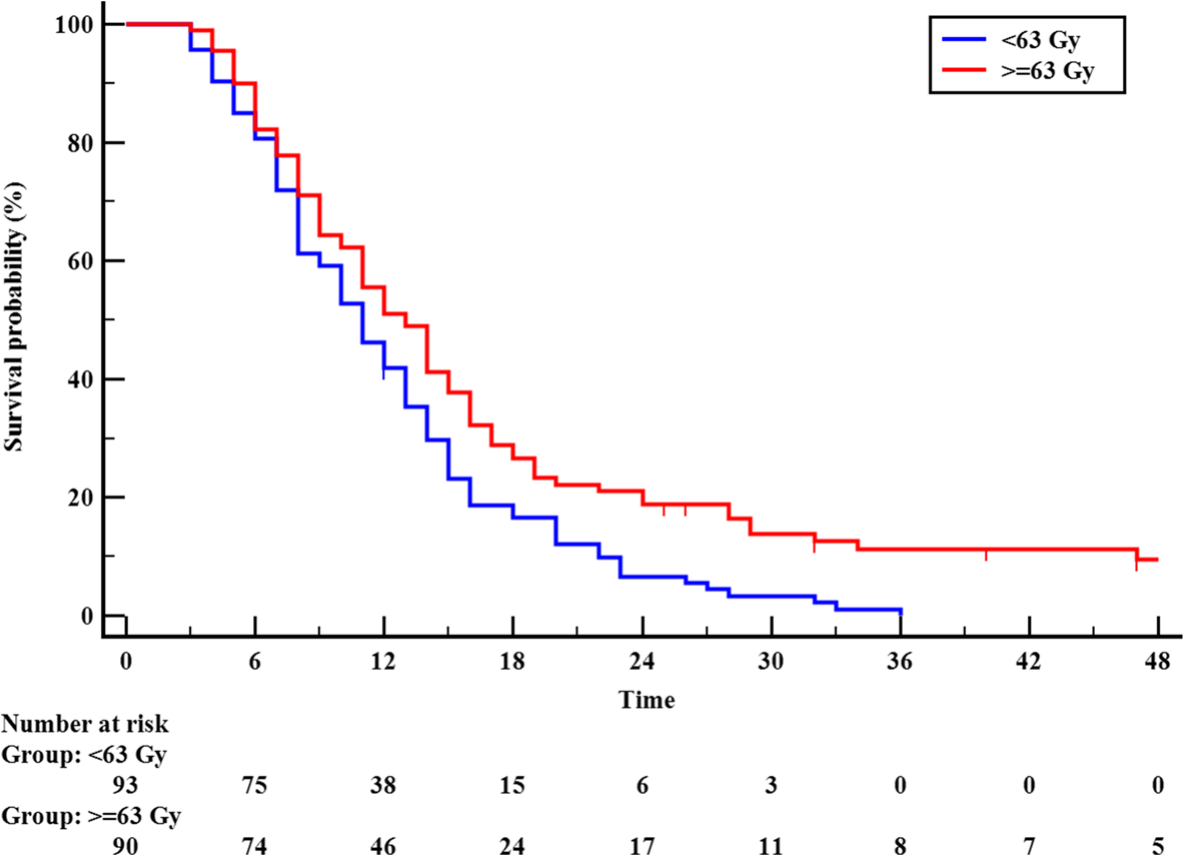
Overall survival in non-oligometastases patients according to radiation dose

At the time of analysis, 41 of 183 non-oligometastatic Stage IV patients died of unknown causes. The cause of death of the remaining142 patients were as follows: most patients died with distant metastasis, only 9 of 142 (6.3%) patients died with primary recurrence alone, 95 (67.0%) patients died with distant metastasis, 13 (9.2%) patients died with distant metastasis and primary recurrence, 12 (8.4%) patients died of other medical disease, 3 (2.1%) patients died with treatment complication, and 10 (7.0%) patients was alive. Univariate analysis showed that radiation dose to the primary tumor (Fig. 2), primary tumor volume, post-treatment KPS score, the number of chemotherapy cycles, and having a treatment response to chemotherapy were significantly associated with OS (Table 2). Multivariate analysis showed that radiation dose, primary tumor volume, post-treatment KPS score, and the treatment response to chemotherapy were significantly associated with OS, as shown in Table 3.

**Table 2:** Univariate analysis for OS in non-oligometastatic patients

Variable	Pre-PSM Cohort					Post-PSM Cohort				
	No	1-yr	2-yr	3-yr	Statistic value	No	1-yr	2-yr	3-yr	Statistic value
Gender										
Male	125	41.6	12.8	4.3	χ^2^ = 1.804	85	36.5	11.8	3.5	χ^2^ = 2.153
Female	58	56.9	12.4	7.8	*P* = 0.179	33	7.6	12.1	6.1	*P* = 0.142
Age (years)										
<60	100	48.0	11.0	3.7	χ^2^ = 0.009	58	44.8	12.1	4.0	χ^2^ = 0.295
≥60	83	44.6	14.9	7.4	*P* = 0.926	60	40.0	11.7	6.7	*P* = 0.587
Tumor histology										
Adenocarcinoma	108	52.8	14.1	6.4	χ^2^ = 2.777	65	41.5	12.3	6.6	χ^2^ = 0.443
Squamous carcinoma	59	37.3	10.2	5.1	*P* = 0.250	44	40.9	9.1	4.5	*P* = 0.801
others	16	37.5	12.5	0.0		9	55.6	11.1	0.0	
Pre-treatment KPS										
70-80	110	49.1	12.0	5.6	χ^2^ = 0.255	64	43.8	7.8	4.7	χ^2^ = 0.001
>80	73	42.5	13.7	4.3	*P* = 0.614	54	40.7	16.7	5.2	*P* = 0.976
T stage										
T1-2	69	50.7	11.9	5.0	χ^2^ = 0.018	41	39.0	9.8	4.9	χ^2^ = 0.862
T3-4	114	43.9	13.2	4.8	*P* = 0.914	77	44.2	13.0	5.4	*P* = 0.353
N stage										
N0-1	25	68.0	21.3	11.3	χ^2^ = 3.696	17	64.7	17.6	17.6	χ^2^ = 3.242
N2-3	158	43.0	11.4	4.5	*P* = 0.055	101	38.6	10.9	3.6	*P* = 0.072
Gross tumor volume, cm^3^										
<146	90	59.2	17.5	6.7	χ^2^ = 7.319	46	50.0	10.9	4.3	χ^2^ = 0.618
≥146	93	37.6	9.7	4.2	*P* = 0.007	72	37.5	12.5	6.2	*P* = 0.432
Post-treatment KPS										
Increased or stable	151	52.3	14.8	5.9	χ^2^ = 15.807	95	47.4	13.7	5.7	χ^2^ = 6.011
Decreased	32	18.8	3.1	3.1	*P* = 0.000	23	21.7	4.3	4.3	*P* = 0.014
Radiation dose, Gy										
≥63	90	51.1	21.1	11.2	χ^2^ = 8.257	59	47.5	20.3	10.8	χ^2^ = 7.013
<63	93	41.9	6.6	0.0	*P* = 0.004	59	37.3	3.4	0	*P* = 0.008
Chemotherapy cycles										
<4	95	37.9	8.7	2.5	χ^2^ = 5.334	60	31.7	6.7	4.4	χ^2^ = 3.775
≥4	88	55.7	1.7	8.3	*P* = 0.021	58	53.4	17.2	6.5	*P* = 0.052
Metastasis status										
Single organ	78	46.2	16.7	8.3	χ^2^ = 1.622	50	46.0	18.0	8.0	χ^2^ = 2.739
2 to 3 organs	105	46.7	9.7	2.9	*P* = 0.203	68	39.7	7.4	3.7	*P* = 0.098
Response to chemotherapy										
No	51	25.5	7.8	3.9	χ^2^ = 10.428	32	21.9	6.2	3.1	χ^2^ = 6.230
Yes	132	54.5	14.6	5.9	*P* = 0.001	86	50.0	14.0	6.1	*P* = 0.013

**Table 3:** Multivariate analyses for OS in non-oligometastatic patient

Variable	Pre-PSM Cohort				Post-PSM Cohort			
	HR	95.0% confidence interval		*P* value	HR	95.0% confidence interval		*P* value
		lower	upper			lower	upper	
Radiation dose, Gy (<63 vs. ≥63)	1.481	1.071	2.047	0.018	1.656	1.125	2.438	0.011
Response to chemotherapy (No vs. Yes)	1.541	1.092	2.176	0.014	1.643	1.073	2.517	0.022
Post-treatment KPS (Decreased vs. Increased or stable)	1.958	1.319	2.907	0.001	1.704	1.064	2.729	0.026
No. of chemotherapy cycles (≥4 vs. <4)	0.796	0.572	1.106	0.173	-	-	-	-
Gross tumor volume, cm^3^ (≥146 vs. <146)	1.479	1.082	2.021	0.014	-	-	-	-

**Fig. 3:**
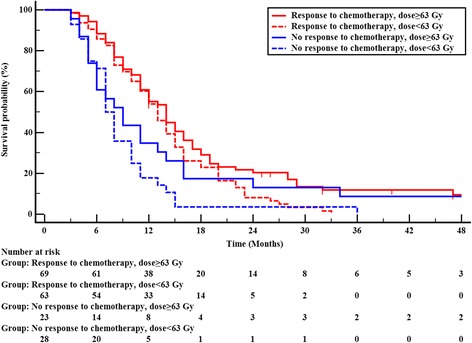
Overall survival according to radiation dose and treatment response of chemotherapy

**Fig. 4:**
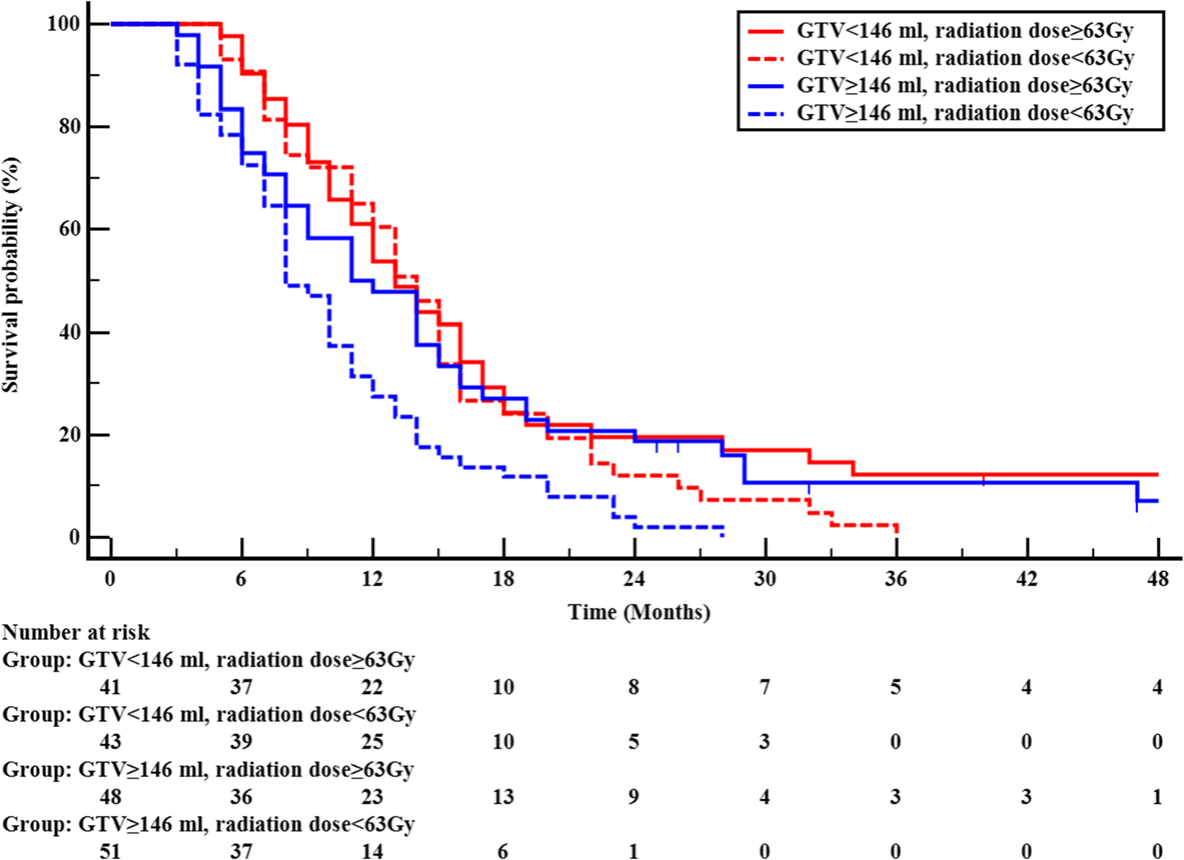
Overall survival according to radiation dose and primary tumor volume

In subgroup analyses, we observed that radiation dose also interacted with treatment response to chemotherapy and primary tumor volume in terms of influencing OS. Total1y, 72.1% (132/183) patients were confirmed to have responded to chemotherapy, and 27.9% (51/183) patients have no response to chemotherapy. Among patients who had a response to chemotherapy, patients who received ≥63 Gy to the primary tumor had a better OS than those received < 63 Gy (χ^2^ = 4.419, *P* = 0.036); patients who had no response to chemotherapy, radiation doses was not correlated with OS (χ^2^ = 1.947, *P* = 0.163), Fig 3. Patients with GTV <146 cm^3^, radiation dose to primary tumor was not associated with OS (χ^2^ = 1.248, *P* = 0.264); among patients with GTV ≥146 cm^3^, a higher radiation dose (≥63 Gy) remained beneficial for OS (χ^2^ = 7.897, *P* = 0.005), Fig 4.

### Propensity score analysis of the impact of radiation dose on OS in non-oligometastatic Stage IV patients

The patient selection factors used to estimate the propensity score were KPS scores, GTV volume, number of chemotherapy cycles and response to chemotherapy. Table 1 summarizes the non-oligometastatic patient characteristics before and after PSM. Before PSM, there were significant differences in pathological type and the number of chemotherapy cycles between the groups that received < 63 Gy and ≥ 63 Gy. After PSM, all clinical characteristic were balanced between the two radiation arms. The 1:1 propensity score–matched cohort consisted of 118 patients with non-oligometastatic disease.

In the post-PSM cohort, radiation dose to the primary tumor, having a treatment response to chemotherapy, and post-treatment KPS score a1so remained to be associated with OS, and the number of chemotherapy cycles had a trend for better OS by univariate analysis (Table 2). On multivariate analysis, these factors retained significance with regard to OS, as shown in Table 3. On landmark analysis for patients surviving at least 8 months, patients who received ≥ 63 Gy to primary tumor retained significance with better OS in Pre-PSM cohort (χ^2^ = 7.953, *P* = 0.005) and post-PSM cohort (χ^2^ = 8.157, *P* = 0.004).

### Survival analysis of oligometastatic Stage IV patients

Among 91 oligometastatic Stage IV patients: most patients died with distant metastasis, only 11 (12.1%) patients died with primary recurrence alone, 43 (47.3%) patients died with distant metastasis, 15 (16.5%) patients died with distant metastasis and primary recurrence, 5 (5.5%) patients died of other medical disease, 12 (13.2%) died of unknown causes, and 5 (5.5%) patients was alive.

Univariate analysis showed that radiation dose to the primary tumor (χ^2^ = 6.150, *P* = 0.013), primary tumor volume (χ^2^ = 5.433, *P* = 0.020), post-treatment KPS score (χ^2^ = 4.730, *P* = 0.030), the number of chemotherapy cycles (χ^2^ = 4.384, *P* = 0.036), and having a treatment response to chemotherapy (χ^2^ = 7.444, *P* = 0.006) were significantly associated with OS. Multivariate analysis showed that radiation dose (*P* = 0.047), and primary tumor volume (*P* = 0.015) predicted OS in these patients with oligometastatic Stage IV NSCLC.

## Discussion

This study sought to investigate whether combining systemic chemotherapy with radiation to the primary tumor could further improve OS of non-oligometastatic Stage IV NSCLC. Compared with historical data [16, 24], this combined therapy in current study produce favorable overall survival. Consistent with previous publication [9], we found that oligometastatic disease and aggressive radiation to the primary tumor were associated with better OS. When the entire group was divided according to metastatic status (oligometastases vs. non-oligometastases), aggressive radiation doses to the primary tumor retained significance for predicting improved survival outcomes.

Consistent with the conclusion of previous studies [7, 9, 10], we found that radiation dose, and primary tumor volume predicted survival in these patients with oligometastatic disease. Among patients with non-oligometastatic disease, defined as ≥5 metastases, we found that receiving higher radiation dose to primary tumor, having a smaller GTV, having response to chemotherapy, and having stable or increased post-treatment KPS scores independently predicted better OS. Most of these predicted factors have been identified in the literature as positive prognostic factors in oligometastatic NSCLC [7–9, 12, 25].

Non-oligometastatic NSCLC patients who are judged to be incurable and have a very short life expectance, radiation is most typically used as palliative treatment when symptoms (hemoptysis, cough, chest pain, dyspnea, and others) emerge. Recent years, there is increasing evidence that selected oligometastatic NSCLC patients could benefit from aggressive thoracic radiotherapy beyond palliative irradiation [7–9, 11, 21, 22]. Comparatively speaking, published studies concerning radiation doses (aggressive or palliative) for non-oligometastatic patients has been limited. In current study, receiving ≥63 Gy to the primary tumor was an independent prognostic predictors of better OS.

Pharmacotherapy has been the main treatment modality, and still play an irreplaceable role for non-metastatic NSCLC. In current study, having response to chemotherapy was an independent prognostic predictors of better OS, and receiving ≥4 cycles chemotherapy was marginally associated with better OS. When the entire group was divided according to response to chemotherapy, higher radiation doses to the primary tumor retained significance for predicting improved survival outcomes in patients who had response to chemotherapy. For the subgroup that had no response to chemotherapy, there was no benefit for improved OS at higher radiation doses. Our findings suggest that non-oligometastatic NSCLC patients benefit from higher radiation doses (≥63 Gy) to the primary tumor based on effective systemic chemotherapy. Thus, in clinical practice, higher radiation dose may be apply in a patient cohorts who have treatment response to effective system therapy. For non-metastatic NSCLC patients who have no response to system therapy, thoracic radiation therapy can be used for palliative intent, whereas, high dose radiotherapy is an unwise choice. In current study, radiation to primary tumor was given concurrently with the chemotherapy. As a result, we recommend further investigation on the value of radiation to primary tumor following effective induction chemotherapy on non-oligometastatic NSCLC.

Recent years, molecular targeted therapy and immunotherapy produce favorable survival outcomes in metastatic NSCLC patients [15, 26, 27]. Because no patients in the current study received molecular targeted therapy or immunotherapy, we cannot comment on whether thoracic radiation combined with molecular targeted therapy or immunotherapy would affect survival. Thus, additional studies are also necessary to investigate the value of thoracic radiation in combination with targeted therapy or immunotherapy for patients with non-oligometastatic NSCLC.

From a clinical standpoint, the larger primary tumor is an indication of a greater tumor burden and source of metastasis, and makes the tumor more difficult to control [28, 29]. Our finding suggest that non-oligometastatic NSCLC patients with smaller primary tumor volume had better OS, consistent with the impact of primary tumor burden on OS for oligometastatic NSCLC [7, 8]. In addition, among patients with larger GTV (≥146 cm^3^), a higher radiation dose (≥63 Gy) remained beneficial for OS; whereas, survival benefit was not observed with higher radiation dose in patients with GTV <146 cm^3^. Our findings suggest that the volume of primary tumor may be used as an indicator to decide radiation dose to primary tumor. We found that stable or increased post-treatment KPS scores were independent predictors of better survival. This finding suggests that post-treatment performance status should be maintained or improved; thus, overtreatment should be avoided when treating non-oligometastatic NSCLC with multimodality therapy.

We acknowledge several limitations of our study. First, consistent imaging data were not gained in a proportion of patients for the evaluation of the relationship between OS and control of primary tumor. Higher radiation doses are associated with improved local tumor control [30]. Although we did not obtain data regarding local control in this study, we speculate that aggressive radiation to primary tumor can improve OS by improving control of primary tumor to reduce the death rate caused by local growth of tumor and decrease the sources of metastasis. Second, the choice of the radiation dose may depend on some factors such as KPS and tumor burden. Although PSM, multivariate regression and landmark analysis were used to reduce this bias, some unaccounted confounders could still have existed between the treatment groups because of the retrospective nature of this study. Therefore, further evidence is needed to confirm conclusions of this study.

## Conclusions

Patients with non-oligometastatic Stage IV NSCLC with good performance status who were treated with aggressive radiation doses (≥63 Gy) to the primary tumor had improved survival outcomes. However, patients benefit from aggressive radiation doses (≥63 Gy) to the primary tumor based on having had response to effective system chemotherapy. Thus, in addition to systemic chemotherapy, we should consider proper radiation dose to primary tumor. Among patients with larger tumors, high radiation dose remained of benefit for OS, and primary tumor volume may be used as an criteria to decide radiation dose. Furthermore, the studies on radiation to primary tumor in non-oligometastatic NSCLC has been limited; and further studies, especially prospective studies, are needed to confirm the outcomes of this treatment modality.

## Abbreviations

2D-RT: Two-dimensional radiotherapy
3DCRT: 3-dimensional conformal radiation therapy
CR: Complete response
GTV: Gross tumor volume
IMRT: Intensity modulated radiation therapy
KPS: Karnofsky Performance Status
NSCLC: Non-small cell lung cancer
OS: Overall survival
PD: Progressive disease
PR: Partial response
PSM: Propensity-score matching
SCLC: Small-cell lung cancer
SD: Stable disease
